# Salvianolic acid B inhibits ototoxic drug–induced ototoxicity by suppression of the mitochondrial apoptosis pathway

**DOI:** 10.1111/jcmm.15345

**Published:** 2020-04-29

**Authors:** Zhiwei Zheng, Yunfeng Wang, Huiqian Yu, Wen Li, Jingfang Wu, Chengfu Cai, Yingzi He

**Affiliations:** ^1^ Department of Otorhinolaryngology Head and Neck Surgery The First Affiliated Hospital School of Medicine Xiamen University Xiamen China; ^2^ Department of ENT institute and Otorhinolaryngology Eye & ENT Hospital State Key Laboratory of Medical Neurobiology Fudan University Shanghai China; ^3^ Teaching Hospital of Fujian Medical University Xiamen China; ^4^ Xiamen Key Laboratory of Otolaryngology Head and Neck Surgery Xiamen China

**Keywords:** HEI‐OC1 cells, ototoxicity, reactive oxygen species, salvianolic acid B, zebrafish

## Abstract

It has been claimed that salvianolic acid B (Sal B), a natural bioactive antioxidant, exerts protective effects in various types of cells. This study aims to evaluate the antioxidant and anti‐apoptosis effects of Sal B in a cultured HEI‐OC1 cell line and in transgenic zebrafish (*Brn3C*: EGFP). A CCK‐8 assay, Annexin V Apoptosis Detection Kit, TUNEL and caspase‐3/7 staining, respectively, examined apoptosis and cell viability. The levels of reactive oxygen species (ROS) were evaluated by CellROX and MitoSOX Red staining. JC‐1 staining was employed to detect the mitochondrial membrane potential (ΔΨm). Western blotting was used to assess expressions of Bax and Bcl‐2. The expression pattern of p‐PI3K and p‐Akt was determined by immunofluorescent staining. We found that Sal B protected against neomycin‐ and cisplatin‐induced apoptotic features, enhanced cell viability and accompanied with decreased caspase‐3 activity in the HEI‐OC1 cells. Supplementary experiments determined that Sal B reduced ROS production (increased ΔΨm), promoted Bcl‐2 expression and down‐regulated the expression of Bax, as well as activated PI3K/AKT signalling pathways in neomycin‐ and cisplatin‐injured HEI‐OC1 cells. Moreover, Sal B markedly decreased the TUNEL signal and protected against neomycin‐ and cisplatin‐induced neuromast HC loss in the transgenic zebrafish. These results unravel a novel role for Sal B as an otoprotective agent against ototoxic drug–induced HC apoptosis, offering a potential use in the treatment of hearing loss.

## 1 INTRODUCTION

Hair cells (HCs) are susceptible to death from various influences including ageing, excessive noise, genetic disorders and ototoxic drugs. Aminoglycoside antibiotics and cisplatin are the two largest classes of valuable clinical drugs with ototoxic adverse effects.^1^, ^2^ Unlike non‐mammalian vertebrates, mammalian cochlear HCs are unable to regenerate once damaged thereby causing permanent hearing loss, which seriously affects the quality of patients’ life. Protection against aminoglycosides and cisplatin‐induced HC damage is therefore an important issue that has attracted a great deal of attention regarding our understanding of the cellular mechanisms underlying these ototoxic drug–induced ototoxicity, and much valuable effort has been focused on identifying novel otoprotective agents.

The amassing of reactive oxygen species (ROS) is considered one major factor underlying aminoglycosides and cisplatin‐induced ototoxicity.^3^ Overproduction of ROS in the mitochondria of HCs overwhelms the redox balance, triggering mitochondrial depolarization and cytochrome* c* release, eventually activating caspase‐3 and inducing apoptosis by modulating various intracellular signalling pathways.^1^, ^4^ Therefore, the application of antioxidants to attenuate free radicals by using ROS scavengers has been currently proposed as promising otoprotectants for mitochondrial function in ototoxic drug–induced ototoxicity. Among various free radical scavengers, natural herbal compounds have attracted consideration, as they are safer and less expensive.

Salvianolic acid B (Sal B) is a major bioactive compound extracted from Radix Salvia miltiorrhiza (Danshen), a popular therapeutic compound used in traditional Chinese medicine as a ROS scavenger to treat various diseases, including hepatitis, menstrual disorders and cardiovascular diseases.^5^, ^6^ Numerous studies have reported that Sal B contains anti‐apoptotic, antioxidant, and anti‐inflammatory properties in vivo and in vitro.^7^, ^8^, ^9^, ^10^ Sal B has been shown to inhibit ischaemia‐reperfusion–induced injury in rat brains by scavenging free radicals and enhancing cerebral energy metabolism.^11^, ^12^ Sal B also exhibited a protective effect against cerebral ischaemia‐reperfusion injury: this may be attributed to the up‐regulation of anti‐apoptotic protein Bcl‐2 expression and maintenance of mitochondrial membrane potential.^13^ However, the result and mechanism of Sal B on aminoglycosides and cisplatin‐mediated ototoxicity is not clearly expounded upon.

This study's aim was to establish whether Sal B possesses a protective action against aminoglycosides and cisplatin‐induced ototoxicity in HEI‐OC1 cell line and in transgenic zebrafish and, if so, the possible mechanisms underlying this action.

## 2 MATERIALS AND METHODS

### 2.1 HEI‐OC1 cell culture and drug treatments

The House Ear Institute‐Organ of Corti 1 (HEI‐OC1) cell line is a widely used auditory HC line derived from the cochlea of the immortomouse.^14^ Cells were grown in acceptable conditions (33°C, 5% CO_2_) in high‐glucose Dulbecco's modified Eagle's medium (DMEM; Gibco BRL) supplemented with 5% foetal bovine serum (FBS; Gibco BRL) without antibiotics. HEI‐OC1 cells were treated with different concentrations of neomycin sulphate (Sigma‐Aldrich, St. Louis, MO, USA, N6386) or cisplatin (Sigma‐Aldrich, 479306) for 24 hours. To investigate the protective efficacy of Sal B (Selleck Chemicals, Houston, TX, USA), HEI‐OC1 cells were pre‐treated with varying concentrations of Sal B for 2 hours and then co‐treated with the optimum concentration of neomycin or cisplatin for 24 hours.

### 2.2 Zebrafish larvae and drug treatments

Zebrafish larvae were raised at 28.5°C in embryo medium with a density of 50 larvae to each 100 mm^2^ Petri dish. Developmental stages were evaluated as days post‐fertilization (dpf). Each zebrafish experiment followed the guidelines of the Institutional Animal Care and Use Committee of Fudan University, Shanghai. For the neomycin studies, 5 dpf larvae were pre‐treated with Sal B for 2 hours and then co‐treated with Sal B and neomycin (200 µM) for another 1 hour. For the cisplatin studies, 4 dpf larvae were incubated for 24 hours with a mixture of 50 μM cisplatin and 40 μM Sal B, following 2 hours pre‐incubation with Sal B. Control animals were exposed to vehicle (DMSO) and included in each experiment.

### 2.3 Immunofluorescence

HEI‐OC1 cells or zebrafish larvae fixed with 4% PFA were then rinsed three times with PBS and permeabilized with 1% Triton X‐100 in PBS (PBST) for 30 minutes. These samples were then blocked with 10% donkey serum in PBST for 1 hour followed by incubation with the anti‐phospho‐AKT at Ser473 (1:500 dilution; Cell Signaling Technology Inc, 9271), anti‐phospho‐PI3 Kinase p85 (Tyr458)/p55 (Tyr199) (1:500 dilution; CST, 4228) or anti‐GFP antibody (1:1,000; Abcam, 13970) overnight at 4°C. The following day, each sample was incubated with the secondary fluorescent antibodies (1:500 dilution; Invitrogen, Carlsbad, Iowa, CA, USA) for 1 hour at 37°C, illuminated. Nuclei were labelled with 4,6‐diamidino‐2‐phenylindole (DAPI; Sigma‐Aldrich, Sigma, D9542) for 10 minutes, illuminated, at room temperature. Each sample was viewed with a Leica SP8 confocal fluorescence microscope (Leica Microsystems).

### 2.4 Cell viability

Cell Counting Kit‐8 (CCK‐8) was employed to gauge cell viability according to the manufacturer's instructions. In brief, HEI‐OC1 cells were seeded at a density of 5000 cells/well in 96‐well plates in three replicates and incubated overnight in acceptable conditions. After drug treatment in 100 μL culture medium, CCK‐8 (Sigma, 96992) was added to each well for 4 hours. The optical density (OD) values were measured at 450 nm using a plate reader (Bio‐Rad). The positive control was subject to the same procedure without cell seeding, while the negative control was left drug‐free. The relative viability was calculated as: (OD_experiment_−OD_positive_)/(OD_negative_−OD_positive_) × 100.

### 2.5 Flow cytometry analysis of apoptosis

Apoptosis was detected from flow cytometry using an Annexin V‐FITC and propidium iodide (PI) kit (BD Biosciences, 556547) according to the manufacturer's instructions. Cells were collected by centrifugation at 3000 × *g* for 5 minutes, washed twice with cold PBS and gently resuspended in 1 × binding buffer at a concentration of 1 × 10^6^ cells/mL. Annexin V‐FITC (5 μL) and PI (5 μL) were introduced, gently mixed with cell suspension and incubated for 15 minutes at illuminated room temperature. The cells were immediately analysed using flow cytometry.

### 2.6 Caspase‐mediated apoptosis assay

HEI‐OC1 cells were cultured in 6‐well plates at a density of 2 × 10^5^ cells per well. Cells after treatment, they were washed in pre‐warmed PBS and stained using 5 μM caspase‐3/7 reagent (Molecular Probes, Life Technologies, United States, C10723) in serum‐free DMEM at 37°C for 30 minutes. Fluorescence microscopy and flow cytometry were used to analyse the fluorescent signal intensity of the cells.

### 2.7 Mitochondrial transmembrane potential measurement

Mitochondrial transmembrane potential (ΔΨm) was estimated by monitoring fluorescence aggregates of JC‐1 (Molecular Probes, Life Technologies, T3168). In brief, HEI‐OC1 cells were seeded in 6‐well plates at a density of 2 × 10^5^ cells/well and subjected to the designate conditions. Cells were washed with pre‐warmed serum‐free DMEM and incubated at 37°C for 30 minutes with 2.5 μg/mL JC‐1. The green fluorescence (JC‐1‐monomer) was viewed at Ex/Em 490/530 nm, while the red fluorescence (JC‐1‐aggregate) was viewed at Ex/Em wavelengths of 525/590 nm. The ratio of the green/red fluorescence (λ530/λ590) indicated mitochondrial depolarization.

### 2.8 TUNEL assay

Apoptosis was determined by TUNEL assay using an in situ cell detection kit (Roche) according to the manufacturer's instructions. Samples were stained with TUNEL reaction mixture at 37°C for 30 minutes in a humid atmosphere, and nuclei were counterstained with DAPI. Viable cells exhibited a normal nucleus and fluorescence in the DAPI channel, whereas dead cells exhibited TUNEL/DAPI double‐positive staining and condensed nuclei. The labelled cells were randomly visualized on a fluorescence microscope at 20 × magnification. The TUNEL/DAPI double‐positive cells were counted using ImagePro Plus image analysis software (Media Cybernetics Inc, Silver Spring), and the number was normalized to the total viable cells to determine TUNEL‐positive rate.

### 2.9 ROS assay

The levels of ROS were detected using CellROX green reagent (Molecular Probes, Life Technologies, USA, C10444) and MitoSOX Red (Molecular Probes, Life Technologies, 1771410). After treatment, samples were washed with pre‐warmed PBS and stained with 5 μM CellROX green for 30 minutes or 5 μM MitoSOX Red 10 minutes.

### 2.10 Protein extraction and Western blot

Cells were lysed with ice‐cold RIPA lysis buffer (Beyotime Institute of Biotechnology, Shanghai, China, P0013B) with protease inhibitor cocktail (Sigma‐Aldrich, P8340) for 30 minutes at 4°C. The lysed cells were centrifuged at 12,000 × *g* for 20 minutes at 4°C. After the supernatant was collected, protein concentrations were detected by the BCA protein assay kit (Beyotime Institute Biotechnology, P0010S). Equal amounts of protein sample were separated by 12% SDS‐PAGE and transferred to polyvinylidene difluoride membranes (Immobilon‐P, Millipore, IPVH00010). The membranes were blocked with 5% non‐fat dried milk in Tris‐buffered saline containing 0.1% Tween‐20 (TBST) for 1 hour at room temperature and incubated with the primary antibodies in TBST containing 5% non‐fat dried milk overnight at 4°C. The primary antibodies were anti‐Bax (1:500 dilution; CST, 2772), anti‐Bcl‐2 (1:1,000 dilution; CST, 3498) and anti‐GAPDH.

### 2.11 FM1‐43FX uptake in zebrafish

Larvae were placed into wells containing the 3 μM FM1‐43FX (Molecular Probes, Eugene, OR, USA) for 45 seconds, illuminated. After being quickly rinsed three times with fresh water, the larvae were anaesthetized and fixed with 4% PFA.

### 2.12 Statistical analysis

All values were shown as mean ± SEM and established through one‐way analysis of variance (ANOVA) or two‐tailed, unpaired Student's *t* test. Statistical analyses were conducted using GraphPad Prism 6 software, with *P* < 0.05 considered statistically significant.

## 3 RESULTS

### 3.1 Sal B protected viability of HEI‐OC1 cells upon neomycin and cisplatin damage

**Figure 1:**
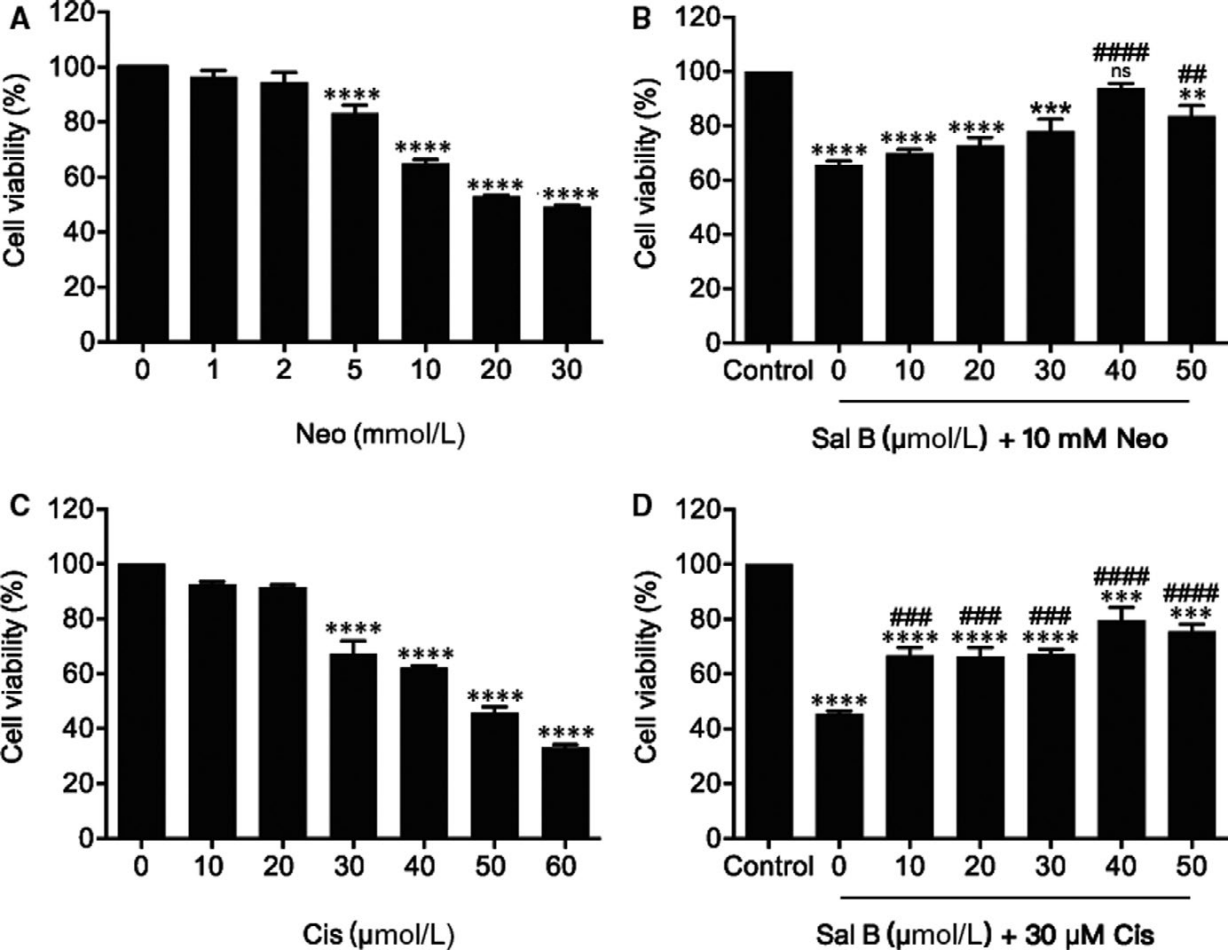
Sal B protected viability of HEI‐OC1 cells upon neomycin and cisplatin toxicity. (A) Effect of neomycin (Neo) on HEI‐OC1 cells. HEI‐OC1 cells were treated with or without various concentrations of Neo (1‐30 mM) for 24 hours. (B) Effect of Sal B on neomycin‐induced viability in HEI‐OC1 cells. Cells were pre‐treated with or without various concentrations of Sal B (10‐50 μM) for 2 hours followed by 10 mM neomycin co‐treatment for 24 hours except those in the non‐treated control (Control) group. (C) Effect of cisplatin (Cis) on HEI‐OC1 cells. (D) Effect of Sal B on cisplatin‐induced ototoxicity in HEI‐OC1 cells. Cell viability was measured by CCK‐8 kit. Values were represented as the mean ± SEM from three independent experiments. **P < 0.01, ***P < 0.001, ****P < 0.0001, and ns. not significant vs the non‐treated control group; ^##^ *P* < 0.01, ^###^ *P* < 0.001 and ^####^ *P* < 0.0001 vs the group treated with 10 mM neomycin or 30 μM cisplatin only

To ascertain the optimal in vitro neomycin ototoxicity model, HEI‐OC1 cells were treated with increasingly concentrated neomycin in the medium (0, 1, 2, 5, 10, 20 or 30 mM) for 24 hours, and their viability was analysed by CCK‐8 assay. As shown in Figure 1A, neomycin treatment significantly reduced the cell viability in a dose‐dependent manner, compared to the non‐treated control group (Figure 1A). Neomycin at a concentration greater than 10 mM markedly reduced cell viability; thus, a neomycin concentration of 10 mM was used for the in vitro study. To determine whether Sal B could protect HEI‐OC1 cells from neomycin‐induced damage, cells were pre‐treated with Sal B concentrations of 0, 10, 20, 30, 40 and 50 μM for 2 hours, and then co‐treated with 10 mM neomycin for 24 hours. We observed a significant protective effect of Sal B at 40 and 50 μM, and a maximal protective effect at a concentration of 40 μM, compared with culture treated with neomycin alone (****P < .0001, **P < 0.01, respectively) (Figure 1B). Notably, Sal B alone (20, 40 and 60 μM) for 24 hours had no significant influence on the viability of HEI‐OC1 cells (Figure S1). These results showed Sal B can protect viability of HEI‐OC1 cells upon neomycin ototoxicity.

To evaluate the protective effect of Sal B against cisplatin‐induced ototoxicity, HEI‐OC1 cells were exposed to increasing concentrations of cisplatin in the culture medium (0, 10, 20, 30, 40, 50 and 60 μM) for 24 hours. As shown in Figure 1C, cisplatin from 30 to 60 μM exposure decreased cell viability from 67.34% ± 4.64% to 33.01% ± 1.18%, compared with the non‐treated control group. Based on the cell viability data, we selected 30 μM cisplatin treatment for 24 hours as the optimal condition for HEI‐OC1 cell injury to the study of cisplatin ototoxicity, as the viability was significantly decreased in comparison with the non‐treated control group. In order to test whether Sal B was able to protect HEI‐OC1 cells from this cisplatin‐induced ototoxicity, the cells were pre‐treated with various Sal B concentrations (0, 10, 20, 30, 40 and 50 μM) for 2 hours, after which they were co‐cultured with 30 μM cisplatin for 24 hours. Using CCK‐8 assay, we confirmed that cell viability in the presence of Sal B was indeed significantly higher than in its absence of Sal B (Figure 1D). Based on these data, we selected 40 μM Sal B, which was associated with maximal cell viability, as the optimal concentration for subsequent experiments. These findings suggested Sal B was able to ameliorate cisplatin‐induced damage to HEI‐OC1 cells.

### 3.2 Sal B protected HEI‐OC1 cells from neomycin‐ and cisplatin‐induced apoptosis

**Figure 2:**
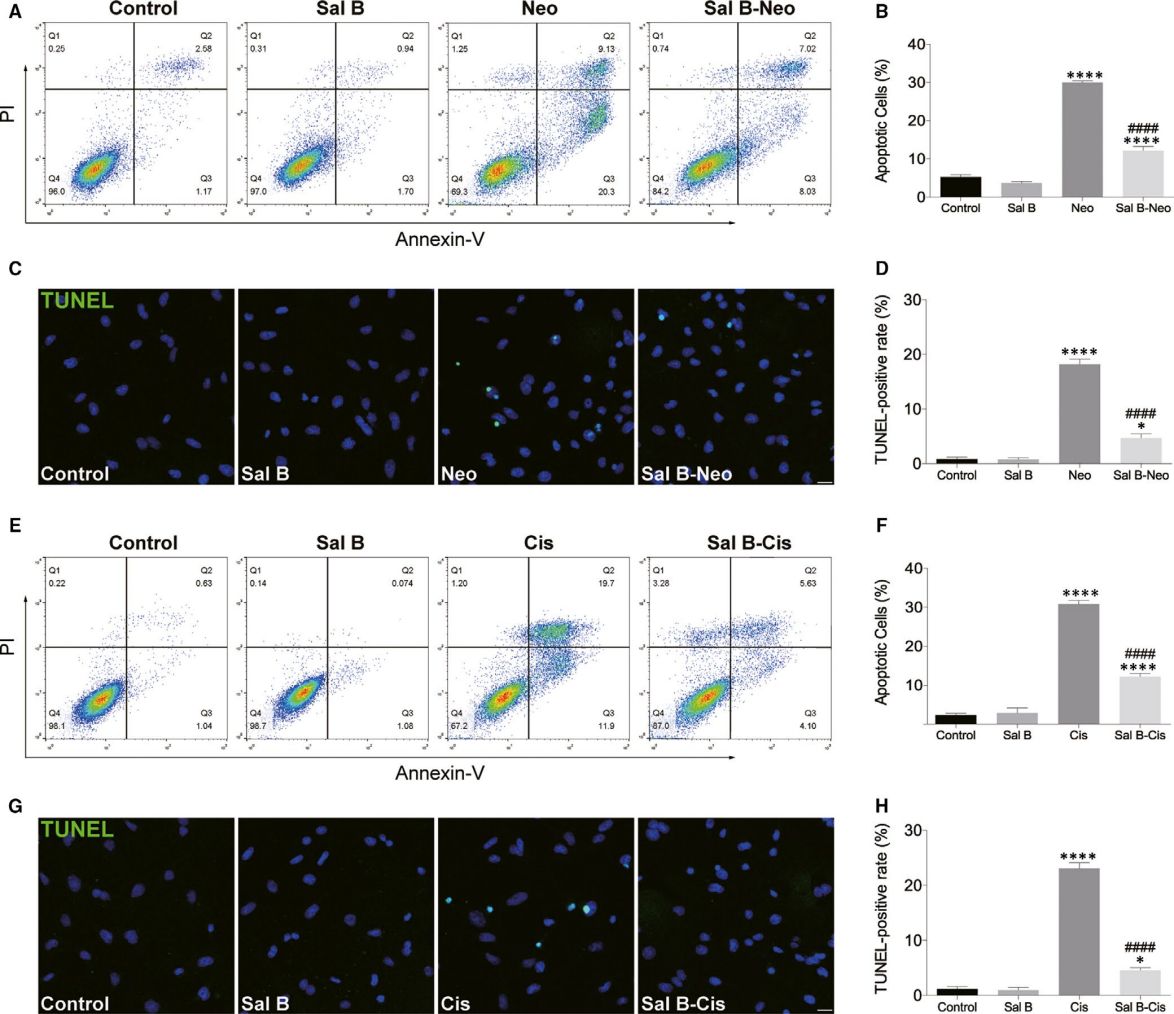
Sal B reduced neomycin‐ and cisplatin‐induced apoptosis in HEI‐OC1 cells. (A and B) HEI‐OC1 cells were pre‐treated with or without Sal B for 2 hours, followed by exposure to 10 mM neomycin for 24 hours except those in the non‐treated control (control) group, and cell apoptosis was determined by flow cytometry using Annexin V and PI staining kit. (C and D) Apoptosis was assessed by TUNEL staining. (E and F) Effect of Sal B on cisplatin‐induced cell apoptosis. (G and H) Apoptosis was assessed by TUNEL staining. Representative images of TUNEL staining from different treatments (G) and quantification of the data (H) were shown. Data were presented as the mean ± SEM. *P < 0.05 and ****P < 0.0001 vs the non‐treated control group; ^####^ *P* < 0.0001 vs the group treated with 10 mM neomycin or 30 μM cisplatin only. Scale bars = 20 μm

To investigate whether the effects of Sal B on HEI‐OC1 cells were due to the reduction of apoptosis, an Annexin V‐FITC and PI double staining was performed by flow cytometry. HEI‐OC1 cells were divided into the following groups: non‐treated control group, 40 μM Sal B treatment group, 10 mM neomycin treatment group, and 10 mM neomycin and 40 μM Sal B co‐treatment group following pre‐treatment with 40 μM Sal B for 2 hours. After 24‐h culture, the cells were double‐stained with Annexin V‐FITC and PI to analyse the percentage of apoptotic cells (Figure 2A). Flow cytometry demonstrated that, after neomycin treatment, cells had significantly greater proportions of apoptotic cells (including late apoptotic cells in the upper right quadrant and early apoptotic cells in the lower right quadrant) than the non‐treated control group. Conversely, Sal B pre‐treatment significantly inhibited neomycin‐induced apoptosis (Figure 2B). The results showed that neomycin promoted HEI‐OC1 cell apoptosis, which was significantly inhibited by Sal B pre‐treatment. To verify these findings, we performed TUNEL staining to detect the apoptotic HEI‐OC1 cells after 10 mM neomycin treatment for 24 hours (Figure 2C). We found a significantly higher percentage of TUNEL‐positive cells in the neomycin alone group, compared with the non‐treated control group. The Sal B pre‐treated group had significantly lower percentages of TUNEL‐positive cells after neomycin damage than the neomycin alone group (Figure 2C,D).

**Figure 3:**
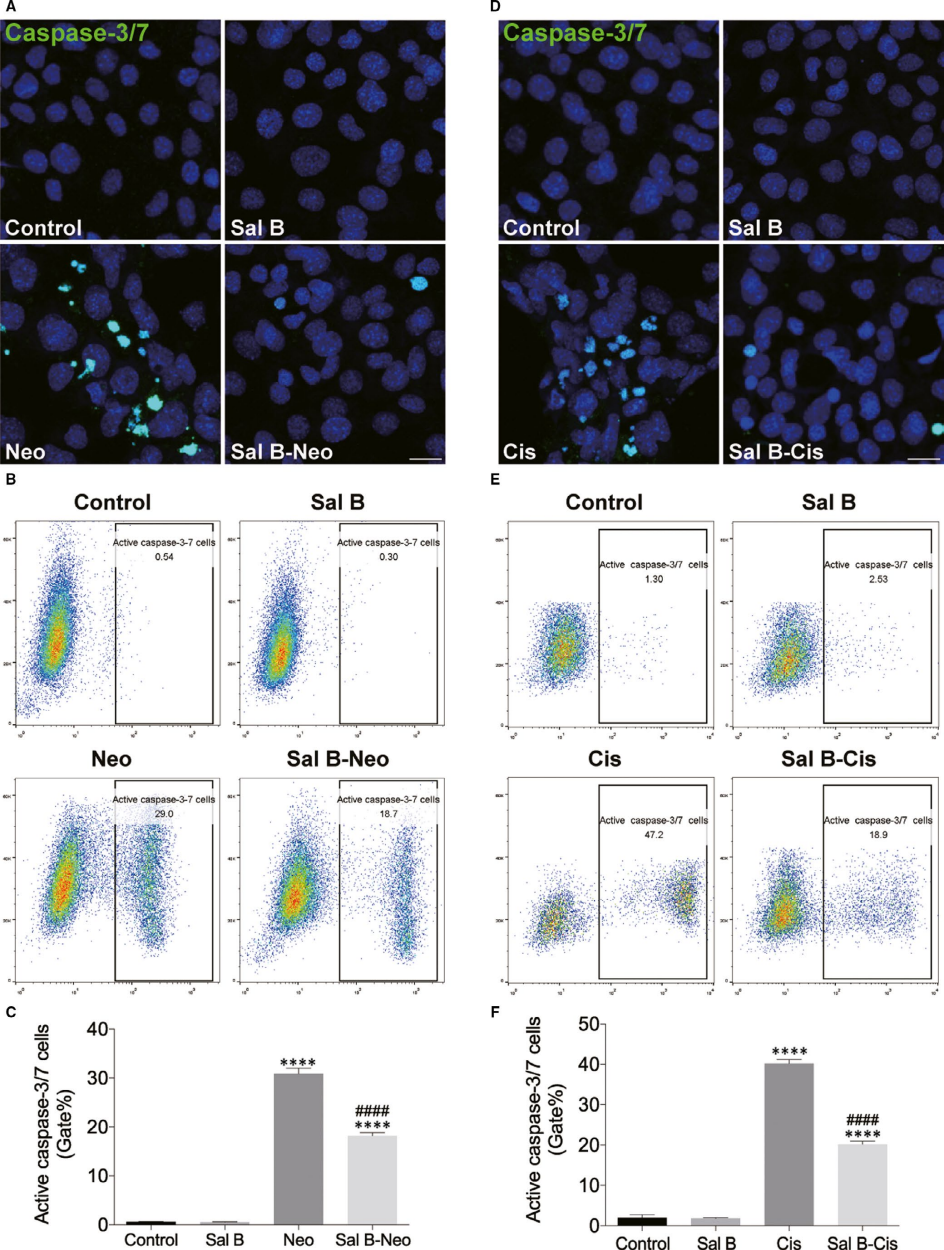
Sal B decreased HEI‐OC1 cell apoptosis after neomycin and cisplatin treatment. (A) Representative images of caspase‐3/7 staining from different treatments in the control, Sal B, neomycin (Neo) and Sal B pre‐treatment after neomycin exposure (Sal B‐Neo). (B) Flow cytometry data confirmed the results in (A). (C) Quantification of the data in (B). (D) Representative images of caspase‐3/7 staining from different treatments in the control, Sal B, cisplatin (Cis) and Sal B pre‐treatment after cisplatin exposure (Sal B‐Cis). (E) Flow cytometry data confirmed the results in (D). (F) Quantification of the data in (E). Data were presented as the mean ± SEM. ****P < 0.0001 vs the non‐treated control group, ^####^ *P* < 0.0001 vs the group treated with 10 mM neomycin or 30 μM cisplatin only. Scale bars = 20 μm

We next performed caspase‐3/7 staining to investigate the underlying molecular mechanism (Figure 3A). Cells in the neomycin alone group showed apoptotic properties, whereas fewer cells displayed these properties in the Sal B pre‐treatment group (Figure 3A). Flow cytometry results showed that Sal B pre‐treated cells had significantly fewer caspase‐3/7–positive cells than the cisplatin alone group (Figure 3B,C).

The impact of Sal B pre‐treatment on cisplatin‐induced cytotoxicity was also examined following Annexin V‐FITC and PI staining. The flow cytometry results showed the proportion of apoptotic cells increased (including early and late apoptotic cells) in the cisplatin alone group compared to the non‐treated control group. In Sal B pre‐treatment group, the proportions of apoptotic cells were significantly smaller those of the cisplatin alone group (Figure 2E,F). We further performed TUNEL assay to evaluate the number of apoptotic cells (Figure 2G,H). As shown in Figure 2G, high TUNEL signal was observed in the cisplatin alone group; however, Sal B pre‐treatment significantly decreased TUNEL‐positive cells and reduced the number of HEI‐OC1 cells displaying the cisplatin‐induced nuclear condensation and apoptotic properties detected by nuclear staining. In addition, the ability of Sal B to decrease apoptotic signals was confirmed by caspase‐3/7 staining. In the cisplatin alone group, the caspase‐3/7 assay showed a high signal that was not seen in the non‐treated control group. By contrast, pre‐treatment with Sal B mostly inhibited caspase‐3/7 expression (Figure 3D‐F). Note that Sal B alone did not cause any damage to the HEI‐OC‐1 cells without neomycin or cisplatin exposure (Figure 2, Figure 3, and Supplemental Figure 1). Therefore, HEI‐OC1 cells were divided into the following groups for the subsequent experiments: control HEI‐OC‐1 cells without any treatment (Control), the cells treated with neomycin (Neo) or cisplatin (Cis) only, and the HEI‐OC‐1 cells treated with Sal B and neomycin (Sal B‐Neo) or cisplatin (Sal B‐Cis).

**Figure 4:**
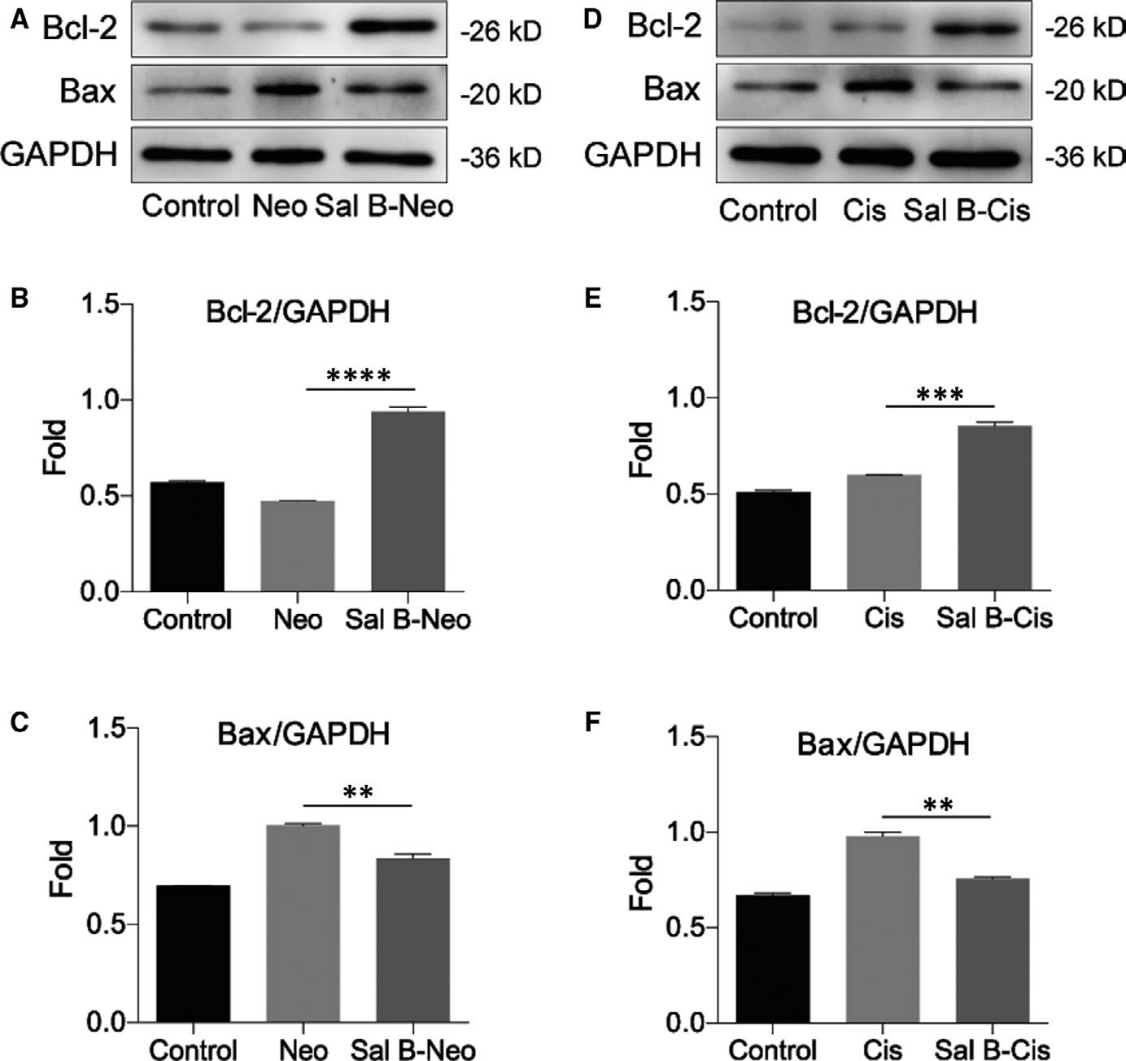
Effect of Sal B on the expressions of the Bcl‐2 family proteins. (A) Representative Western blot image showed the expression of Bcl‐2 and Bax in the control, Neo and Sal B‐Neo groups, and the columns (B and C) showed the quantification of the fold changes of expression. (D) Representative Western blot image showed the expression of Bcl‐2 and Bax in the control, Cis and Sal B‐Cis groups, and the columns (E and F) showed the quantification of the fold changes of expression. The data expression for protein was normalized by GAPDH. Data were presented as the mean ± SEM. **P < 0.01, ***P < 0.001, ****P < 0.0001

Using Western blot, we examined the expressions of the Bcl‐2 family proteins, finding neomycin exposure‐induced cell apoptosis through the intrinsic pathway, demonstrated by elevated mitochondria‐dependent pro‐apoptotic protein Bax compared with the non‐treated control group. In comparison, pre‐treatment with Sal B markedly reduced the expression of Bax and promoted anti‐apoptotic protein Bcl‐2 expression comparing to the cells that were exposed to neomycin alone (Figure 4A‐C). Moreover, the expression of Bax was attenuated, and the expression of Bcl‐2 markedly increased in the group pre‐treated with Sal B following cisplatin exposure in comparison with the cisplatin alone group (Figure 4D‐F). These new data suggested that Sal B possessed the capability to inhibit ototoxic drug–induced intrinsic apoptotic pathway in HEI‐OC1 cells.

### 3.3 Sal B reduced neomycin‐ and cisplatin‐induced ROS generation and increased mitochondrial membrane potential in HEI‐OC1 cells

**Figure 5:**
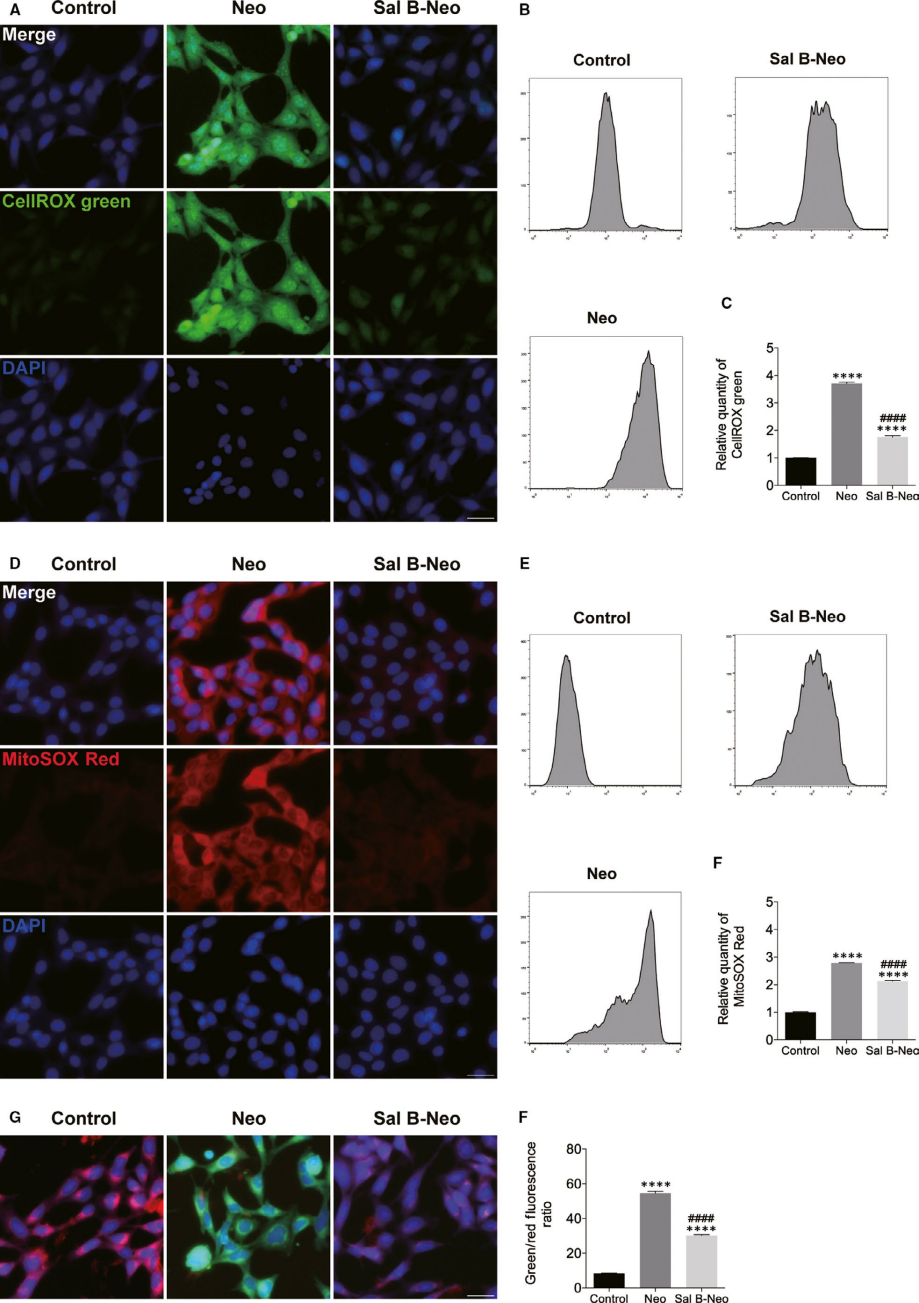
Sal B reduced ROS production and increased mitochondrial membrane potential (ΔΨm) in neomycin‐injured HEI‐OC1 cells. (A‐F) HEI‐OC1 cells were pre‐treated with 40 μM Sal B for 24 hours prior to being exposed to 10 mM neomycin. The levels of intracellular and mitochondrial ROS were evaluated using CellROX green (A‐C) and MitoSOX Red staining (D‐F). (A) Representative images of CellROX green staining in the control, neomycin (Neo) and Sal B pre‐treatment after neomycin exposure (Sal B‐Neo). (B) Flow cytometry data confirmed the results in (A). (C) Quantification of the data in (B). (D) Representative images of MitoSOX Red staining from different treatments. (E) Flow cytometry data confirmed the results in (D). (F) Quantification of the data in (E). (G and H) A mitochondrial membrane potential assay kit was used to measure ΔΨm. (G) Representative images of JC‐1 staining in the control, neomycin (Neo) and Sal B pre‐treatment after neomycin treatment (Sal B‐Neo). (H) Quantification of the fluorescent signal intensity in (G). Data were presented as the mean ± SEM. ****P < 0.0001 vs the non‐treated control group; ^####^ *P* < 0.0001 vs the group treated with neomycin only. Scale bars = 20 μm

Neomycin was reported to cause the overproduction of radical oxygen species (ROS), which is integral to apoptosis as it activates multiple apoptotic pathways.^15^ We investigated whether Sal B was able to inhibit this process. Cells from different groups were collected and stained with ROS indicator dye CellROX green. As expected, neomycin resulted in a noticeable elevation of green fluorescence of CellROX in HEI‐OC1 cells, compared with the non‐treated control group. Conversely, pre‐treatment with Sal B significantly inhibited ROS production induced by neomycin exposure (Figure 5A‐C). We next monitored mitochondrial ROS using the probe MitoSOX Red in HEI‐OC1 culture cells from different treatments. In accordance with CellROX green results, the MitoSOX Red fluorescence was strongly diminished in Sal B pre‐treated cells, indicating the protective effect of Sal B from neomycin damage was associated with the inhibition of generation ROS (Figure 5D‐F). Excessive ROS are able to interrupt the mitochondrial membrane potential (ΔΨm), which is considered one of the earliest hallmarks of mitochondrial dysfunction, which ultimately leads to apoptosis.^16^ ΔΨm was assessed using the JC‐1 staining. As shown in Figure 5G, HEI‐OC1 cells exposed to neomycin condition for 24 hours resulted in decreased mitochondrial membrane potential compared with the non‐treated control group. This is demonstrated by the increased ratio of green to red fluorescence (Figure 5G,H). Pre‐treatment with Sal B significantly increased ΔΨm in neomycin‐damaged HEI‐OC1 cells (*P* < 0.0001).

**Figure 6:**
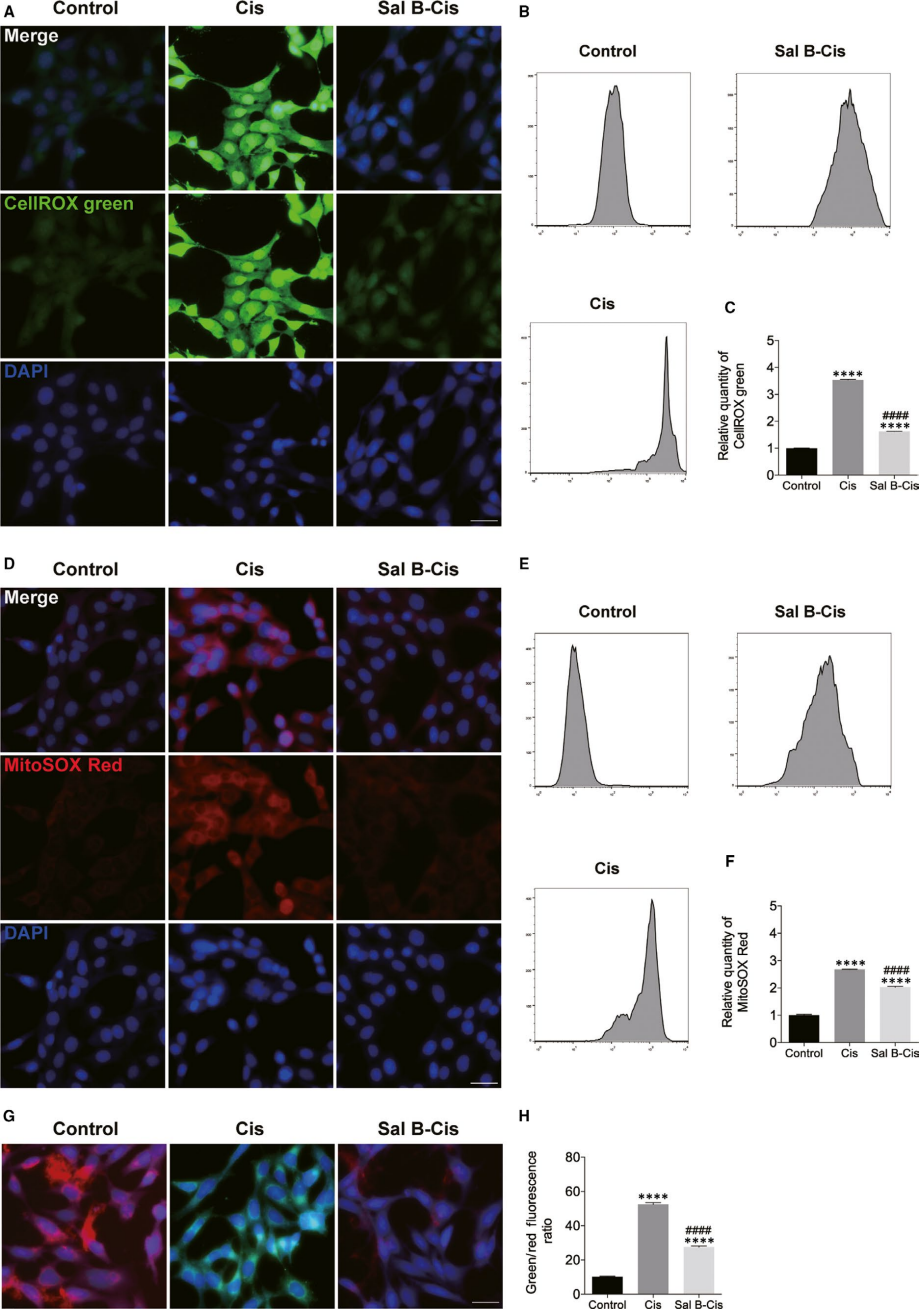
Sal B reduced ROS production and increased ΔΨm in cisplatin‐injured HEI‐OC1 cells. (A‐F) Effect of Sal B on ROS production in cisplatin‐damaged HEI‐OC1 cells. (G and H) Effect of Sal B on ΔΨm in cisplatin‐injured HEI‐OC1 cells. Data were presented as the mean ± SEM. ****P < 0.0001 vs the non‐treated control group; ^####^ *P* < 0.0001 vs the group treated with cisplatin only. Scale bars = 20 μm

To examine whether Sal B pre‐treatment inhibited the cisplatin‐induced generation of ROS, the levels of intracellular and mitochondrial ROS were measured by CellROX green and MitoSOX Red staining, respectively. In the Sal B followed by the cisplatin exposure group, a significant decrease in green fluorescence (CellROX green) or red fluorescence (MitoSOX Red) was observed compared to that of the cisplatin only group (Figure 6). These results showed that Sal B significantly decreased cisplatin‐induced elevation of ROS. Next, the effects of cisplatin and the cisplatin/Sal B combination on ΔΨm were examined as a marker of mitochondrial function. The mitochondrial membrane potential change induced by cisplatin treatment and the protective effect of Sal B was deduced from the ratio of green and red fluorescence. As demonstrated in Figure 6G, 30 μM cisplatin induced a significant reduction in ΔΨm following treatment for 24 hours compared with the normal control. Treatment of the cells with a 40 µm concentration of Sal B and a 30 µm concentration of cisplatin significantly prevented the reduction of ΔΨm compared with the cells treated with cisplatin alone (Figure 6G,H). Collectively, these results suggested ototoxic drug–induced mitochondrial dysfunction in HEI‐OC1 cells, and Sal B was able to protect mitochondrial function.

### 3.4 Sal B attenuated neomycin‐ and cisplatin‐induced cytotoxicity through activation of the PI3K/AKT pathway

**Figure 7:**
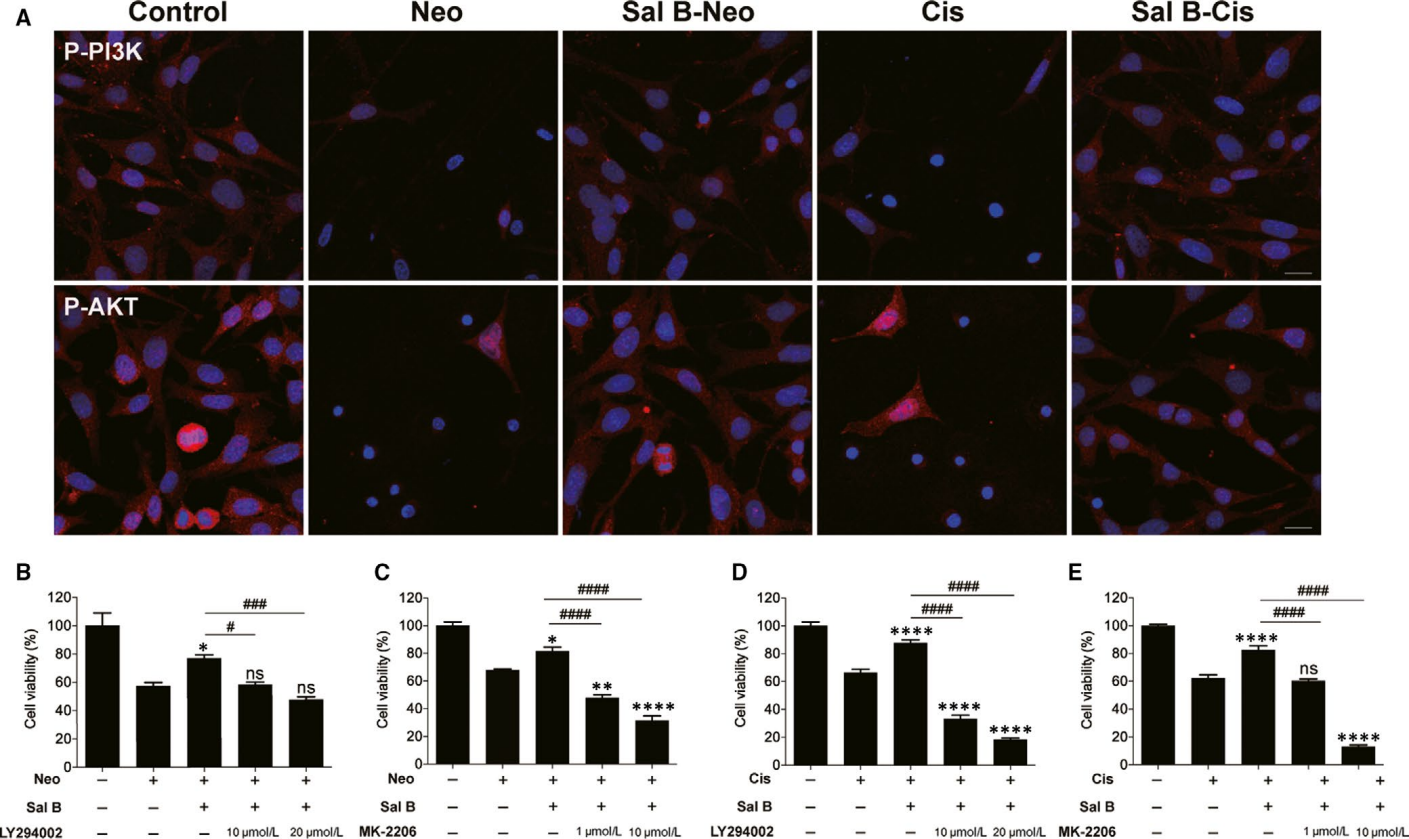
Sal B activated the PI3K/AKT pathway in HEI‐OC1 cells undergoing neomycin‐ and cisplatin‐induced apoptosis. (A) Effect of Sal B on the expression of phospho‐PI3K (P‐PI3K) and phospho‐AKT (P‐AKT) in neomycin‐ and cisplatin‐damaged HEI‐OC1 cells. (B‐E) HEI‐OC1 cells were pre‐treated with Sal B (40 μM) and LY294002 (10 or 20 μM) or MK‐2206 (1 or 10 μM) for 2 hours and then exposed to 10 mM neomycin or 30 μM cisplatin for 24 hours. Cell viability was quantified by CCK‐8 assay. Data were presented as the mean ± SEM. *P < 0.05, **P < 0.01, ****P < 0.0001 and ns. not significant vs the group treated with 10 mM neomycin or 30 μM cisplatin only; ^#^ *P* < 0.05, ^###^ *P* < 0.001 and ^####^ *P* < 0.0001 vs the group treated with Sal B and neomycin or cisplatin. Scale bars = 20 μm

Accumulating evidence indicates that many signalling pathways such as phosphatidylinositol 3‐kinase (PI3K)/AKT/mTOR^17^ and mitogen‐activated protein kinase kinase (MEK)/extracellular signal‐regulated kinase (ERK)^18^, ^19^ are involved in mediating HC survival. We examined a possible link between the anti‐apoptotic effects of Sal B and PI3K/AKT or MEK/ERK pathways. Western blotting analysis suggested that ototoxin (neomycin or cisplatin) significantly decreased the expression of P‐PI3K and P‐AKT, but did not affect the degree of phosphorylation of ERK (Figure S2), and Sal B treatment increased the expression of P‐PI3K and P‐AKT (Figure S2). Immunostaining analysis confirmed that the phosphorylation of PI3K and AKT was weakly positive in the neomycin or cisplatin group compared to the control group after 24 hours, while the phosphorylation of the PI3K and AKT in Sal B plus ototoxin (neomycin or cisplatin) groups was significantly increased compared with that of the neomycin or cisplatin alone group (Figure 7A). To further determine the involvement of PI3K/AKT pathway in Sal B‐induced protection, HEI‐OC1 cells were treated with LY294002 (a PI3K inhibitor) or MK‐2206 (a specific AKT inhibitor). Treatment with LY294002 and MK‐2206 selectively decreased P‐PI3K and P‐AKT levels in a dose‐dependent manner (Figure S3). CCK‐8 was used to analyse the cell viability changes after inhibitor treatment. Results showed that inhibition of PI3K/AKT with LY294002 or MK‐2206 attenuated the increase in cell viability induced by Sal B in a dose‐dependent manner (Figure 7B‐E). Additionally, co‐exposure to PD98059, a MEK inhibitor, did not attenuate the Sal B‐induced cytoprotective effect (Figure S4). These results indicated the mechanisms of Sal B‐induced protective effects could be mediated by activating the PI3K/AKT pathway in ototoxin‐treated HEI‐OC1 cells.

### 3.5 Sal B protected against neomycin‐ and cisplatin‐induced HC death in zebrafish lateral line

**Figure 8:**
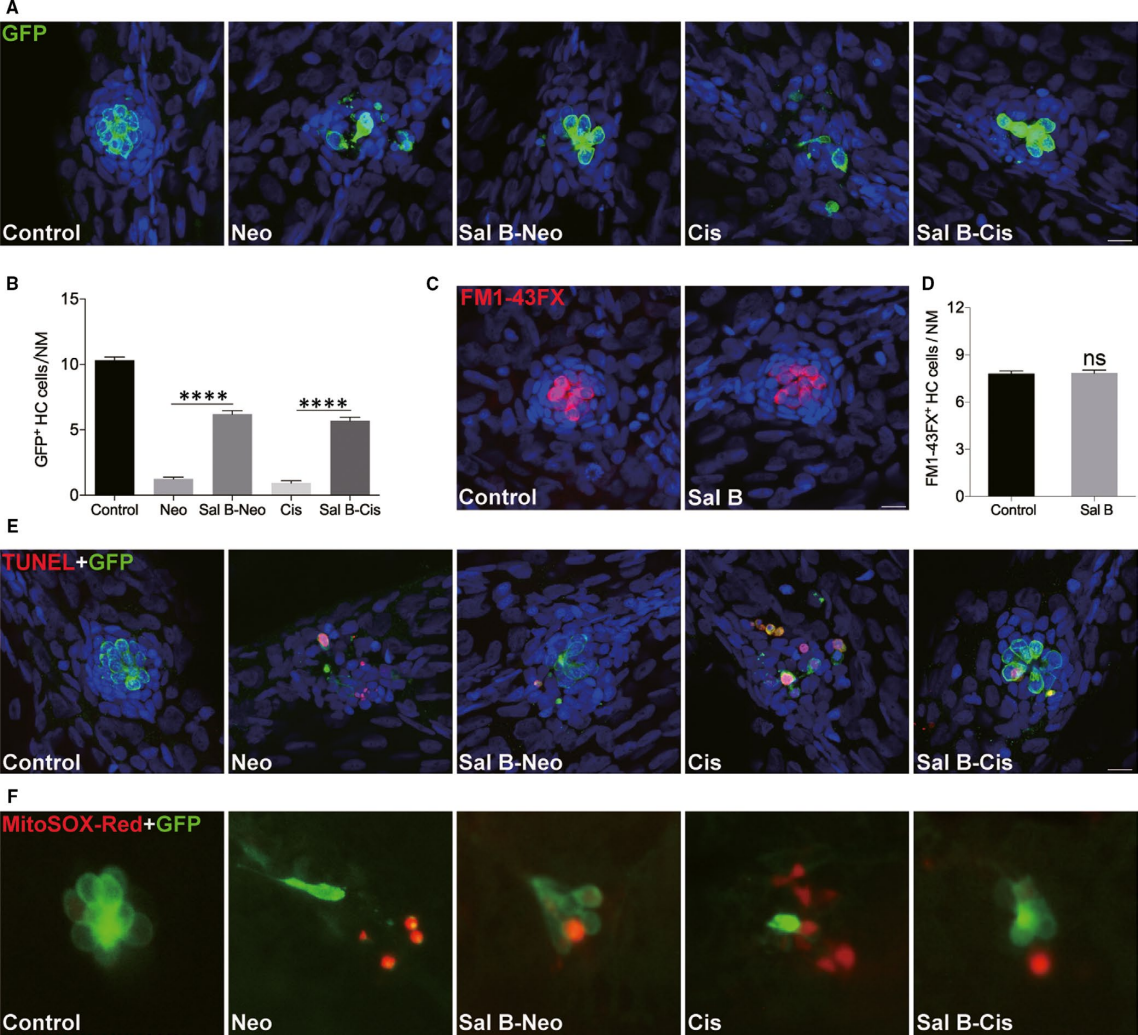
Sal B protected against neomycin‐ and cisplatin‐induced neuromast HC loss in the transgenic zebrafish. (A and B) Effect of Sal B on hair cell loss induced by neomycin and cisplatin. Hair cells were imaged by GFP staining. Representative images of GFP immunostaining from control, Neo, Sal B‐Neo, Cis and Sal B‐Cis groups (A) or of the data (B) were shown. (C and D) Mechanotransductively active hair cells were assessed by FM1‐43FX staining. (E) Apoptosis was assessed by TUNEL assay. Apoptotic cells are marked as light red dots. (F) Detection of ROS with MitoSOX Red staining. Data were presented as the mean ± SEM. ****P < 0.0001; ns. not significant. Scale bars = 10 μm

To investigate the protective effect of Sal B in vivo, a transgenic zebrafish line *tg* (*Brn3C*: EGFP), which express membrane‐bound green fluorescent protein in hair cells under the control of the *brn3c* promoter, was used. We first examined Sal B’s potential effect on neomycin toxicity. Larvae treated with neomycin alone showed a significant HC loss compared to that of control animals (Figure 8A,B). However, pre‐treatment of zebrafish larvae with 40 μM Sal B for 2 hours, followed by co‐treatment with 200 μM neomycin for 1 hour, showed protection of neuromast HCs compared to that of the neomycin alone (Figure 8A,B). Next, we studied Sal B’s potential otoprotective effect on cisplatin by pre‐treating larval zebrafish at 4 dpf with Sal B concentration of 40 µM for 2 hours followed by co‐treatment with 50 µM cisplatin for 24 hours. As shown in Figure 8A, 24‐h exposure of 50 µM cisplatin resulted in a significant loss of HCs in the neuromast of zebrafish larvae compared to that of the control animals. Conversely, Sal B could significantly ameliorate the HC loss induced by cisplatin. Previous work has shown that aminoglycosides and cisplatin can enter the HCs through the mechanotransduction channels. We therefore decided to address whether Sal B protected from ototoxic damage by blocking the mechanotransduction (MET) channels. The fluorescent vital dye FM1‐43FX, a marker for functional MET channels in hair cells,^20^ was used in control larvae and larvae exposed to Sal B 40 μM for 2 hours. Figure 8C shows that rapid dye entry into the HCs was comparable between the control and Sal B treatment groups. Quantification of the FM1‐43FX‐positive cells did not show any significant differences (Figure 8D), indicating that Sal B did not affect the MET activity.

The TUNEL assay was conducted to ascertain whether ototoxin induced the death of neuromast cells by apoptosis, and whether that apoptosis could be prevented by Sal B treatment. As seen in Figure 8E, while the ototoxin alone treatment group showed more incidents of cell apoptosis compared with that of the undamaged control group, the Sal B pre‐treatment group showed significantly reduced incidents of apoptosis compared those treatments with ototoxin alone. As aminoglycosides’ and cisplatin's ototoxic effects are associated with ROS accumulation, MitoSOX Red staining was performed to evaluate ROS generation (Figure 8F). Increased staining was detected in the neuromast of ototoxin alone treated larvae; however, little staining was observed in the Sal B pre‐treatment group. These results suggested administration of Sal B limited the generation of ROS in response to ototoxin damage.

## 4 DISCUSSION

Sal B, bioactive component purified from the roots of Salvia miltiorrhiza Bge, is a traditional Chinese medicine extensively utilized as a powerful ROS scavenger to treat varying diseases like cardiovascular, hepatitis and menstrual disorders.^5^, ^6^ Sal B has been stated to have the anti‐apoptotic and antioxidant effects.^7^, ^8^, ^21^ In addition, multiple studies have reported that Sal B has neuroprotective effects on many central nervous system disorders.^7^, ^21^, ^22^ However, no study has yet investigated the possible benefits of Sal B in ototoxic insult–induced ototoxicity. This study showed that Sal B offered an important protective effect against neomycin‐ and cisplatin‐induced ototoxicity in both in vivo and in vitro experiments. The protective effects of Sal B have been shown here by CCK‐8 assay in maintaining the viability of neomycin‐ and cisplatin‐insulted HEI‐OC1 cells, and demonstrated by reducing Annexin V‐FITC/PI staining. Our results established that otoprotection in response to Sal B was caused by the prevention of cellular ROS production and inhibition of the intrinsic caspase‐3 apoptotic pathway. This study has revealed for the first time that Sal B might serve as a potential otoprotective agent for protecting against ototoxic insult–induced hearing loss.

Aminoglycosides and cisplatin were widely reported to induce intrinsic (mitochondrial death) apoptosis of HCs through oxidative stress, eventually causing ototoxicity.^23^, ^24^ The mitochondria‐mediated apoptotic pathway is regulated by the combined actions of the pro‐ and anti‐apoptotic members of the Bcl‐2 family proteins. Caspase‐3 is the final effector in the mitochondria‐mediated apoptosis.^25^ Our results showed that exposure to ototoxic insults induced a significant up‐regulation of the pro‐apoptotic proteins and down‐regulation of the anti‐apoptotic protein Bcl‐2, whereas Sal B effectively reversed these effects. This finding was congruous with the beneficial effect of Sal B on HEI‐OC1 cell apoptosis, indicating Sal B can stabilize the mitochondria and alleviate the activation of the mitochondrial apoptotic pathway, thereby protecting cells from neomycin‐ and cisplatin‐induced apoptosis.

It has been documented that increased production of intracellular ROS level is a major mechanism underlying ototoxic insult–induced HC death.^26^, ^27^ Excessive ROS violates proper action of the HCs, reducing antioxidant defence, triggering release of apoptosis‐related factor cytochrome *c* from mitochondria and caspase‐3 pathway activation and leading to apoptosis.^28^ To determine whether Sal B exerted its protective functions against neomycin and cisplatin through alleviating cellular oxidative stress, we therefore examined the production of ROS by performing a cellular ROS assay and a mitochondria‐specific ROS indicator MitoSOX Red. Our results indicated that Sal B significantly decreased the production of cellular ROS induced by neomycin and cisplatin in HEI‐OC1 cells, which in turn greatly attenuated apoptosis. Our observations were consistent with Sal B being an ROS scavenger,^11^, ^12^ which indicated that Sal B antioxidative property might be a major mechanism underlying the anti‐apoptotic effects of Sal B on HCs.

It is well known that HC apoptosis is the major mechanism underlying the aminoglycoside‐ and cisplatin‐induced ototoxicity and ROS‐associated mitochondrial damage is involved in the hair cell apoptosis. Currently, antioxidants and anti‐apoptotic agents have been shown to interfere with ROS pathways and to prevent HC loss in mammals.^29^ In this study, we demonstrated that administration of Sal B dramatically decreases the mitochondrial ROS and prevents mitochondrial dysfunction and apoptosis of HEI‐OC‐1 cells after ototoxic drug injury. Note that although Sal B treatment significantly attenuates neomycin‐ or cisplatin‐induced injury, there is still a loss of HCs compared to undamaged controls. Thus, exploring more effective mitochondria‐targeted antioxidants that can be coadministrated with Sal B will be critical to maximize the protective effect of Sal B to rescue HC damaged by ototoxic drugs. Apart from the inhibition of mitochondrial apoptotic pathway, hair cell survival is also dependent upon multiple mechanisms. For example, recent work has shown that autophagy activation is an important contributor to attenuate the neomycin‐ or cisplatin‐induced ototoxicity by suppressing ROS accumulation.^30^, ^31^, ^32^ Additionally, our previous study showed that G9a inhibitor and some LSD1 inhibitors could protect against ototoxic drug–induced HC death, suggesting that epigenetic mechanisms are involved in ototoxic drug–induced hearing loss.^33^, ^34^ The present study focuses on the mitochondrial apoptotic pathway in neomycin‐ or cisplatin‐induced HC injury; no other mechanisms or their crosstalk was analysed. Thus, more complete picture of molecular and epigenetic pathways should be elucidated in future work by use of appropriate pharmacologic or genetic approaches.

Increasing evidence has suggested that overproduction of ROS can stimulate numerous intracellular signalling pathways, such as PI3K/AKT and MEK/ERK pathways.^17^, ^35^ PI3K/AKT is known to have potential anti‐apoptotic functions through several downstream targets including phosphorylation of Bad (serine‐136) and has recently been shown to be involved in promoting hair cell survival.^17^ Our present findings demonstrated that Sal B did not notably affect the phosphorylation of ERK; it seems possible that ERK activation might not be the direct targets of Sal B. Additionally, our data demonstrated that exposure to Sal B could lead to an increased phosphorylation of PI3K and AKT after ototoxic drug treatment. These findings indicate that the anti‐apoptotic effect of Sal B on ototoxic drug–induced ototoxicity might be partly attributed to the restoration of activation of PI3K/AKT signalling. Future research to investigate the precise mechanisms that underlie the otoprotective effect of Sal B in HCs will shed more light on our understanding of HC survival molecular mechanisms. Hence, these results might be important in future clinical settings. Even with consideration of these studies, application to auditory HCs of mammals still requires investigation. Therefore, additional studies should occur to determine the protective effect of Sal B in the mammalian inner ear.

**Figure 9:**
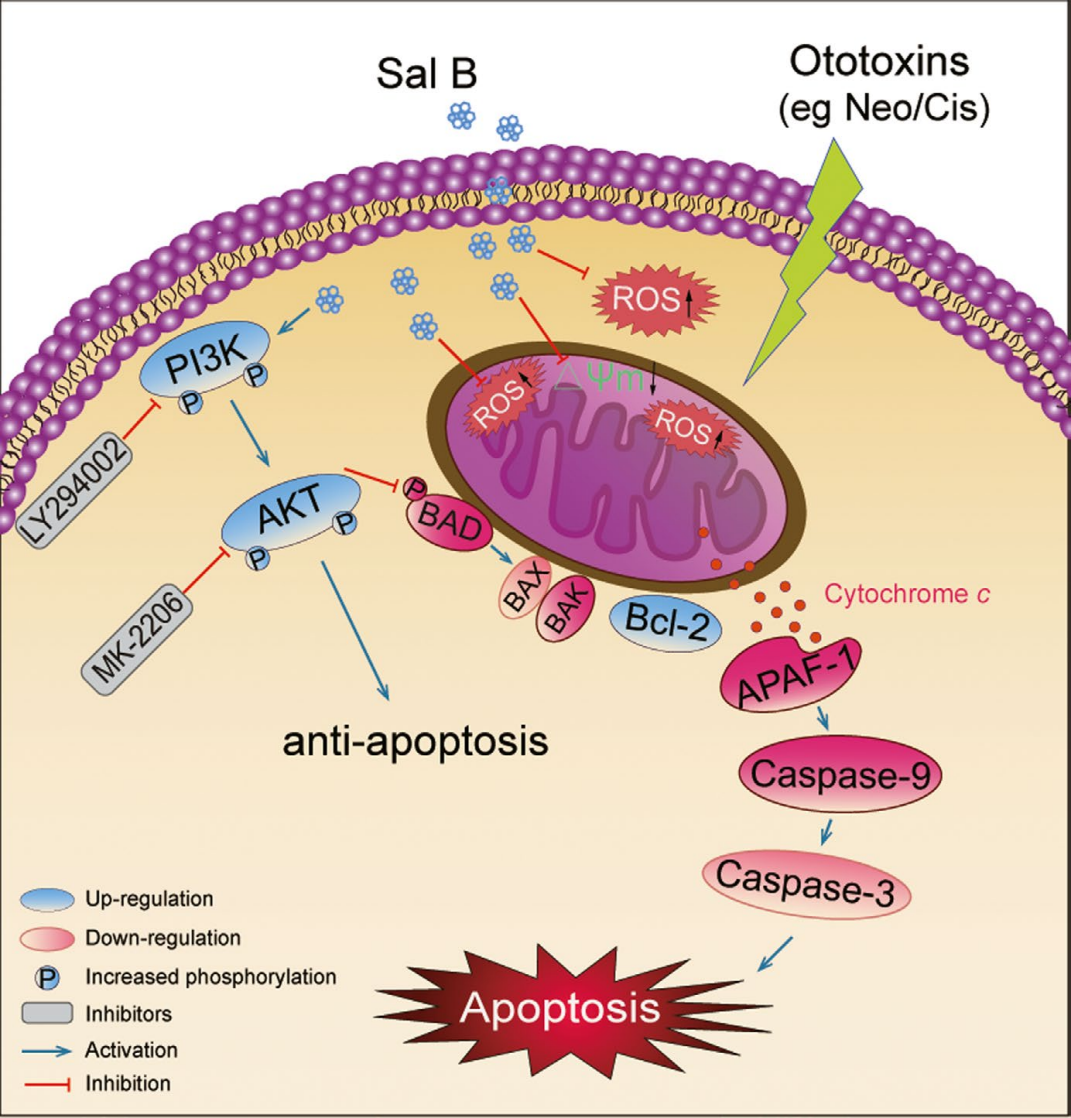
Schematic illustrating the possible mechanisms of protective effect of Sal B against ototoxic drug–induced apoptosis. As illustrated, neomycin or cisplatin treatment markedly induces the production of ROS and triggers mitochondrial apoptotic pathway. Sal B treatment could reduce the generation of ROS; decrease the expression levels of apoptosis proteins; increase the expression levels of anti‐apoptotic proteins; induce the activation of anti‐apoptotic PI3K/AKT pathway; and eventually ameliorate the oxidative stress–induced apoptosis

In summation, we demonstrated that Sal B exhibited significant effect on protection against aminoglycoside antibiotics and cisplatin‐induced ototoxicity by suppressing the production of ROS and mitochondrial apoptotic pathway (as summarized in Figure 9). Our findings have yielded an initial instance indicating Sal B as a preventive or therapeutic agent in the treatment of ototoxic insult–induced hearing loss.

## CONFLICT OF INTEREST

The authors declare no competing financial interests.

## AUTHOR CONTRIBUTIONS

ZZ, YW, CC and YH designed the study. ZZ, YW, HY, WL and JW performed experiments. HY, CC and YH analysed data. CC and YH wrote the manuscript. ZZ, YW, HY, WL, JW, CC and YH commented on the manuscript.

## Supporting information

Fig S1

Fig S2

Fig S3

Fig S4

## Data Availability

The data that support the findings of this study are available from the corresponding author upon reasonable request.
