# Supplementary figures and images for: Salvianolic acid B inhibits ototoxic drug–induced ototoxicity by suppression of the mitochondrial apoptosis pathway
Source: J Cell Mol Med. 2020 Apr 29;24(12):6883–97. doi: 10.1111/jcmm.15345 (PMC7299715; doi:10.1111/jcmm.15345)

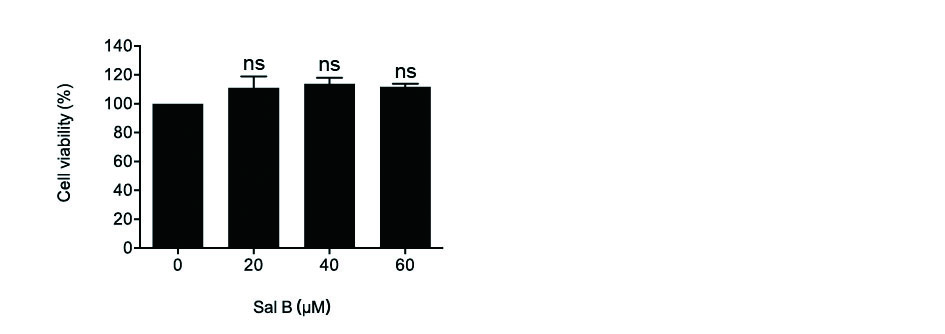

Supplement: Supplementary file 1 — Fig S1 [file JCMM-24-6883-s001.tif]

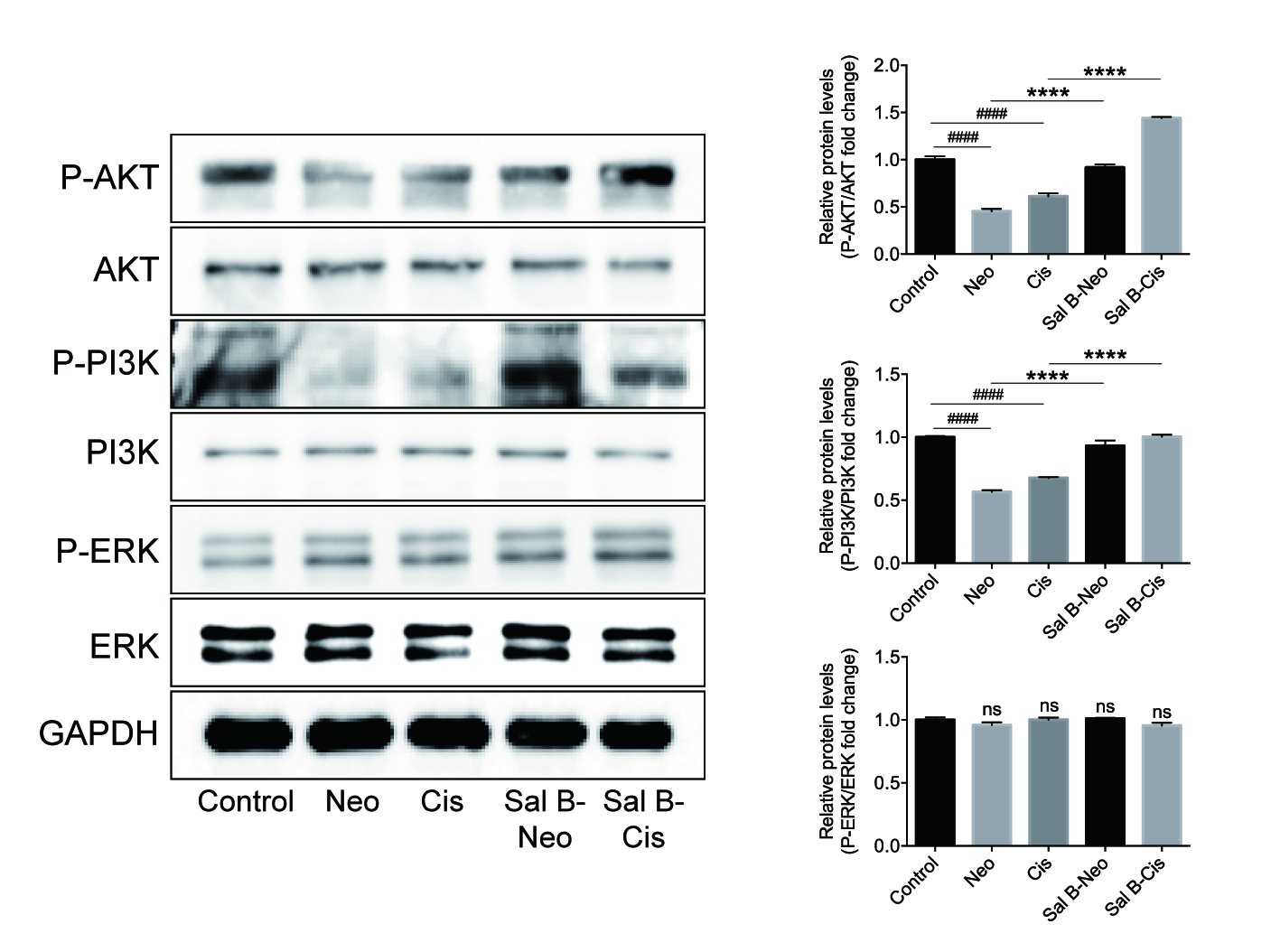

Supplement: Supplementary file 2 — Fig S2 [file JCMM-24-6883-s002.tif]

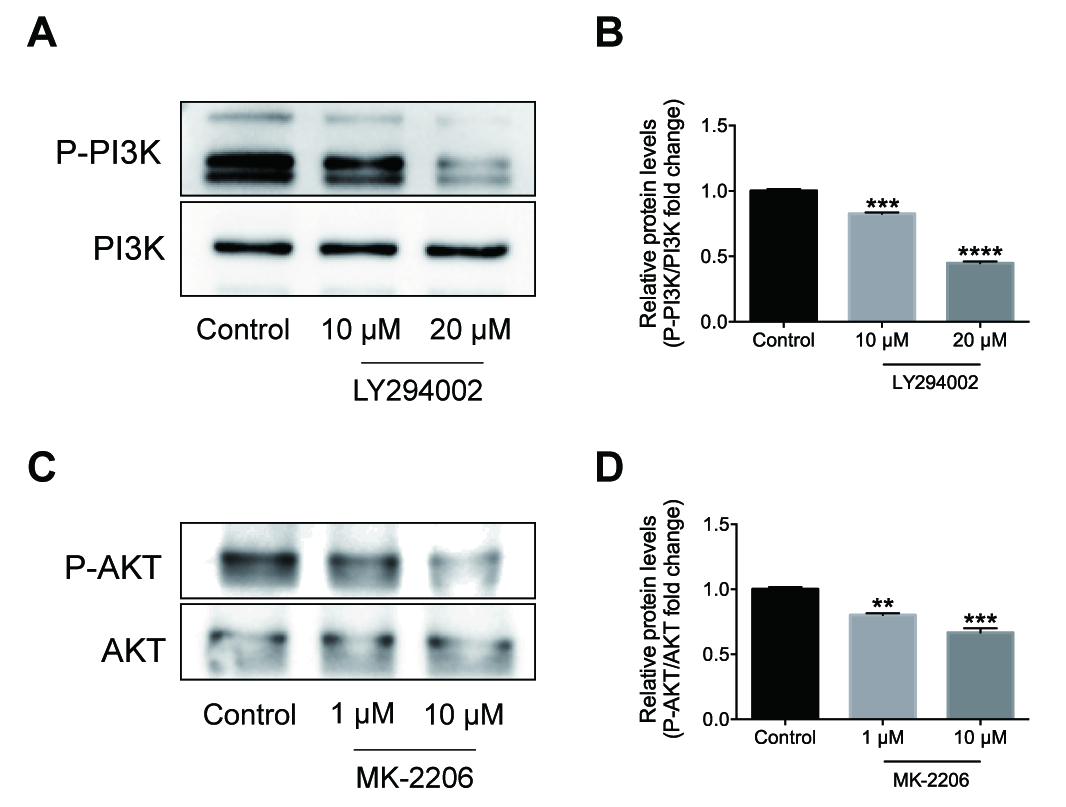

Supplement: Supplementary file 3 — Fig S3 [file JCMM-24-6883-s003.tif]

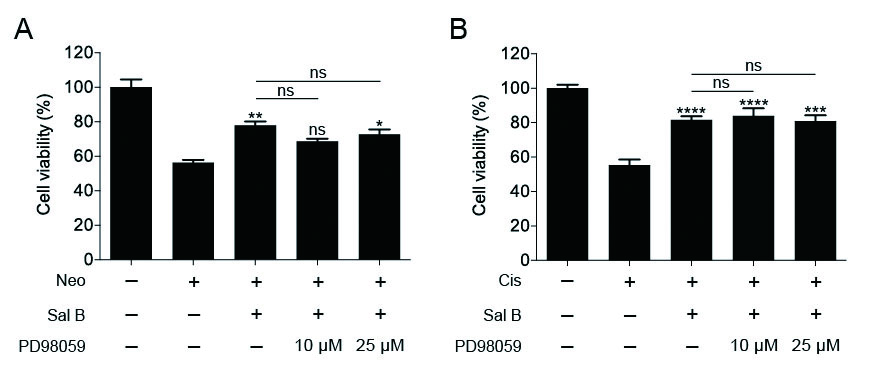

Supplement: Supplementary file 4 — Fig S4 [file JCMM-24-6883-s004.tif]
